# Incidence of malnutrition, esophageal stenosis and respiratory complications among children with repaired esophageal atresia

**DOI:** 10.1590/0102-672020190001e1486

**Published:** 2020-11-13

**Authors:** Shahnam ASKARPOUR, Mehran PEYVASTEH, Mozhgan DASHTYAN, Hazhir JAVAHERIZADEH, Mitra AHMADI, Mohsen ALI-SAMIR

**Affiliations:** 1 Nursing Care Research Center in Chronic Diseases; 2 Department of Pediatric Surgery; 3 Student Research Committee; 4 Department of Pediatric Gastroenterology; 5 Alimentary Tract Research Center, Ahvaz Jundishapur University of Medical Sciences, Ahvaz, Khouzestan, Iran

**Keywords:** Esophageal stenosis, Esophagus, Malnutrition, Estenose esofágica, Esôfago, Desnutrição

## Abstract

**Background:**

Esophageal atresia is congenital anomaly with high mortality. Surgical complications and changes in nutritional status are common problems after surgical correction.

**Aim: T:**

o evaluate nutritional status, esophageal stenosis, and respiratory complications among children who had repaired esophageal atresia.

**Methods:**

Children aged >2 months old with repaired esophageal atresia were included in the current study. Gender, age, weight, and height were recorded for each case. Height for age and weight for age were calculated for each case.

**Results:**

According to weight for length percentile, 41.02% of the cases were underweight. Esophageal stenosis was seen in 54.76% of the obtained esophagograms.

**Conclusion:**

Underweight was present in 41.02 of the patients according to weight-for-height percentile.

## RESUMO

**Racional:**

Atresia de esôfago é anomalia congênita com mortalidade alta. Complicações cirúrgicas e alterações no estado nutricional são problemas comuns após correção cirúrgica.

**Objetivo:**

Avaliar o estado nutricional, a estenose esofágica e as complicações respiratórias em crianças que tiveram a reparação de atresia de esôfago.

**Métodos:**

Crianças com idade> 2 meses de idade com atresia esofágica reparada foram incluídas no estudo atual. Sexo, idade, peso e altura foram registrados para cada caso. A altura por idade e o peso por idade foram calculados para cada caso.

**Resultados:**

De acordo com o peso para o percentil de comprimento, 41,02% dos casos estavam abaixo do peso. Estenose esofágica foi observada em 54,76% do esofagograma obtido.

**Conclusão:**

O baixo peso esteve presente em 41,02 dos pacientes, de acordo com o percentil peso/estatura.

## INTRODUCTION

Esophageal atresia is a congenital anomaly with estimate prevalence about 1/2500-3000 live births^1^. Mortality on it has decreased from 75% to 58% in our setting^2^. So, complication of repaired esophageal atresia and nutritional status of the living child is the most important problem in our hospital. 

There are few published researches with the focus on nutritional status among the children with repaired esophageal atresia. The aim of this study was to evaluate malnutrition rate among children with repaired esophageal atresia.

## METHODS

This study was cross sectional and retrospective. It was approved by the institutional Ethics Committee under number IR.AJUMS.REC.1396.57

Hospital charts of the Department of Pediatric Surgery of Imam Khomeini Hospital and outpatient clinic of Abuzar children’s hospital of children aged >2 months old with repaired esophageal atresia were reviewed. Gender, age, weight, and height were recorded for each case. Height and weight for age were calculated for each case. Percentile of weight for height for boys and girls were calculated (Table 1). For children under three years recumbent position was used for length measurement. Infant weight for age and child weight for age were also calculated for each case. 

**TABLE 1:** Classification of malnutrition according to weight for height percentile

Percentile <5	Underweight
Percentile >=5 and <85	Healthy weight
Percentile >=85 and <95	At risk of overweight
Percentile >=95	Overweight

## RESULTS

Of 43 children, 25 were male and 18 female. In 39, recorded mean birth body weight was 2914 g (1800-4500). According to weight for length percentile, 41.02% of our cases were underweight (Table 2).

**TABLE 2:** Result of weight for height (length) among children with repaired esophageal atresia

Percentile	n=39
Percentile <5 (underweight)	16(41/02%)
Percentile >=5 and <85 (healthy weight)	14(35/90%)
Percentile >=85 and <95 (at risk of overweight)	5(12/83%)
Percentile >=95 (overweight)	4(10/25)

Among 41 records about respiratory problem, 14 (34.14%) children had persistent problem. Among 42 recorded data about contrast esophagograms, 23 children had esophageal stenosis; seven normal esophagus. Contrast esophagogram was not done for 12 due to esophageal stenosis. As a result esophageal stenosis was seen in 54.76% in esophagograms.

## DISCUSSION

Male was slightly more affected than female with esophageal atresia^3^ ^-^ ^5^, which is similar to the current study. Our results showed, according to weight for length percentile, undernutrition present among 41.02% of children with repaired esophageal atresia. In another study from China on 10 patients with esophageal atresia, mild malnutrition was seen in five and severe in one^6^. Undernutrition in our study was slightly lower than that study^7^. This high rate of undernutrition may be due to the high frequency of undernutrition in our country^7^ ^,^ ^8^.

Respiratory complications which was seen in 34.14% of the cases are multifactorial and may be due anastomotic leaks^9^ ^,^ ^10^, recurrence of fistula, and anastomotic stricture. Tracheomalacia was seen in 37.5% to 75% of the children who underwent surgery of esophageal atresia^11^ ^,^ ^12^. Another reason for high rate of respiratory problems may be due to gastroesophageal reflux disease^13^. Esophageal stenosis following repair of esophageal atresia and/or trachea-esophageal fistula was seen in 54.76% of the obtained contrast esophagogram.

## CONCLUSION

In repaired esophageal atresia malnutrition was seen in 41.02%, esophageal stenosis in 54.7% and respiratory problems in 34.14% of the cases.
